# Prolonged Rapamycin treatment led to beneficial metabolic switch

**DOI:** 10.18632/aging.100554

**Published:** 2013-05-04

**Authors:** Yimin Fang, Andrzej Bartke

**Affiliations:** Geriatrics Laboratory, Department of Internal Medicine, Southern Illinois University School of Medicine, Springfield, IL 62794, USA

The evolutionarily conserved TOR (Target of Rapamycin) signaling controls growth and metabolism from yeast to mammals [1]. Mammalian or mechanistic TOR (mTOR) plays the key role in aging and age-related disease [2]. Rapamycin, a drug used clinically for organ transplants, coronary stent coating and certain forms of cancer treatments, is an inhibitor for mTOR. In the first robust demonstration of pharmacologically-induced life extension in a mammal, rapamycin increased longevity of mice via either feeding or injection [2]. However, rapamycin treatment also showed the detrimental metabolic effects, including hyperinsulinemia, hyperlipidemia, glucose intolerance and insulin resistance. Those observations present a paradox of improved survival despite metabolic impairments. How rapamycin extended lifespan with such paradoxical metabolic effects remains to be elucidated [3]. In the various studies of rapamycin treatment, length of rapamycin treatment varied from two weeks to two years. With short-term rapamycin treatment, mice showed the detrimental metabolic effects, while a much longer length (up to 1.5 to 2 years) of rapamycin treatment led to increased longevity. Duration of rapamycin treatment may be one of the key factors that determine outcomes of the treatment. Longer-term rapamycin treatment may cause beneficial metabolic “switch” that is associated with enhanced insulin signaling and extended longevity.

In the issue of Cell Metabolism (Volume 17, Issue 3, 456-462, 5 March 2013), we reported that duration of rapamycin treatment indeed has differential effects on metabolism. In our study, rapamycin was given to mice for two, six or 20 weeks. Consistently with the previous reports, mice with two weeks of rapamycin treatment had characteristics of metabolic syndrome. Mice with six weeks of rapamycin treatment were in the metabolic transition status. When rapamycin treatment continued for 20 weeks, the detrimental metabolic effects were reversed or diminished. Insulin signaling is important in the control of longevity in both mice and humans. Lower insulin levels and higher insulin sensitivity are associated with extended longevity in long-lived mutants, such as Ames dwarf or GHR-KO (Growth Hormone Receptor Knock-out) mice [4]. In our study, alterations in insulin sensitivity induced by different durations of rapamycin treatment were closely associated with changes of glucose and lipid homeostasis and metabolism, as well as body composition. Short-term rapamycin treatment increased insulin levels drastically, but reduced insulin sensitivity with lower insulin signaling represented by lower phosphorylation of AKT at Ser473 (a key phosphorylation site to activate AKT), lower insulin tolerance and higher HOMA-IR (Homeostatic Model for Assessment of Insulin Resistance) scores. Additionally, pancreas mass was decreased and liver mass was increased, two body characteristics associated with metabolic syndrome. It is worth mentioning in a recent study, which has shown that “prolonged” rapamycin treatment caused insulin resistance, the mice were treated with rapamycin for 2 to 4 weeks [5]. This could reflect the effects of “short”-term rapamycin treatment in our study. Therefore, it is not surprising to observe insulin resistance after 2 to 4 weeks of rapamycin treatment in liver Rictor knock-out mice [5]. 20 weeks of rapamycin treatment decreased insulin levels, but enhanced insulin sensitivity significantly. Most likely due to hypoinsulinemia, mice with 20 weeks of rapamycin treatment had higher fed glucose and a certain degree of glucose intolerance in the early stage of GTT (Glucose Tolerance Test), however, those mice had normal glucose levels during fasting, suggesting hypersensitivity to insulin, and ITT (Insulin Tolerance Test) results showed enhanced insulin sensitivity as well. Additionally, levels of Grb10, a newly-identified insulin signaling inhibitor which lies downstream of mTOR [6] were decreased in the muscle from mice with 20 weeks of rapamycin treatment (unpublished data). Consequently, mice eventually were able to clear glucose, albeit at a slower pace due to lower basal levels of insulin and higher insulin sensitivity, and HOMA-IR was much lower than in the controls. Lipid metabolism was also altered in relation to the length of rapamycin treatment. Mice with 20 weeks of rapamycin treatment had reduced adiposity and better lipid profiles with increased oxygen consumption (one of indicators of more lipid usage as fuel), and enhanced ketogenesis (a process that is involved in fatty acid breakdown and linked to modulation of aging). Interestingly, similar to the findings in human renal transplant patients, who received rapamycin as an immunosuppressant for 12 months, hypertriglyceridemia detected after short rapamycin treatment was normalized. mTOR inhibits insulin-induced stimulation of lipogenesis and enhances insulin-regulated lipolysis [7]. Higher insulin levels after short-term rapamycin treatment may cause impairment of normal flux of lipid metabolism by enhancing lipogenesis and inhibiting lipolysis, indicated by higher levels of triglycerides and lower levels of glycerol. After 20 weeks of rapamycin treatment, insulin levels dropped substantially. Theoretically, lipogenesis should be lower and lipolysis should be higher, generating more glycerol and NEFA (Non-Esterified Fatty Acids). However, instead of increasing, both glycerol and NEFA, especially NEFA, were decreased. These changes suggest that some lipid metabolic processes, such as using more fatty acids as fuel, could be triggered and enhanced. Consequently, energy metabolism in mice with 20 weeks of rapamycin treatment switched from low (using more carbohydrates as metabolic substrates) to high (expending more energy to burn more fatty acids), most likely via enhancing uncoupled energy generation processes (unpublished data). Taken together, prolonged rapamycin treatment caused beneficial metabolic switch, possibly by increasing metabolic flexibility [8] triggered by mTOR controlled insulin-induced lipid metabolism, which in turn may enhance insulin sensitivity in glucose metabolism.
